# A LOOK AT STUDENT CAREGIVING EXPERIENCES: OPPORTUNITIES FOR CAMPUSES AND COMMUNITIES

**DOI:** 10.1093/geroni/igae098.1570

**Published:** 2024-12-31

**Authors:** Allyson Graf, Katherina Nikzad-Terhune, Joseph Gaugler

**Affiliations:** Chair; Co-Chair; Discussant

## Abstract

Caregiving in the United States continues to be a significant and growing phenomenon that warrants ongoing exploration of ways to support caregivers. Further examining the impact that intergenerational connections and age-inclusive practices have on the caregiving experience can help inform ways for both campuses and communities to be more responsive to caregiver needs. This symposium features participants representing universities that have capitalized on the Age-Inclusivity in Higher Education (AIHE) model to generate new and creative forms of caregiver support and intergenerational connections. These research endeavors represent the distinct ways that the AIHE model can be utilized to recognize and respond to the diverse needs of caregivers on campus and in the community. Terhune/Graf/Manning (Northern Kentucky University) will discuss mixed-methods data on student caregivers and how this knowledge has informed new student caregiving initiatives both on campus and with community partners. Hawes (University of Wisconsin-Eau Claire) will present a synthesis of intergenerational programs conducted in nursing homes between 2020 and 2024. Drawing from 25 student applied projects in select facilities, the research aims to identify best practices and challenges with implementing intergenerational efforts in nursing homes. Elfenbein (University of North Georgia) will present information about ACAPcommunity and ACAP Hall County, bringing students and community caregivers together to learn about caregiving issues and resources, and support one another on the caregiving journey. Graf/Baker/Bartlett (Northern Kentucky University) will elaborate on different ways that caregiving experience may associate with outlooks on caregiving, personal aging and health by comparing current, former, and indirect caregivers.

